# Role of Hypothalamus in Acupuncture’s Effects

**DOI:** 10.3390/brainsci15010072

**Published:** 2025-01-15

**Authors:** Ryan Bae, Hyung Kyu Kim, Baoji Lu, Jing Ma, Juping Xing, Hee Young Kim

**Affiliations:** 1 Morrissey College of Arts and Sciences, Boston College, Boston, MA 02467, USA; 2 Department of Physiology, College of Medicine, Yonsei University, Seoul 03722, Republic of Korea

**Keywords:** acupuncture, lateral hypothalamic area, nucleus accumbens, dopamine, orexin, hypocretin, hypothalamic–pituitary adrenal axis, neuropeptide Y, AgRP, pain, stress, anxiety, addiction

## Abstract

The significant correlation between ancient medicinal practices and brain function marks a revolutionary frontier in the field of neuroscience. Acupuncture, a traditional oriental medicine, can affect brain regions, such as the hypothalamus, anterior cingulate, posterior cingulate, and hippocampus, and produces specific therapeutic effects, such as pain relief, suppression of hypertension, and alleviation of drug addiction. Among the brain regions, the hypothalamus, a small yet critical region in the brain, plays a pivotal role in maintaining homeostasis by regulating a wide array of physiological processes, including stress responses, energy balance, and pain modulation. Emerging evidence suggests that acupuncture may exert its therapeutic effects by modulating the activity of the hypothalamus and its associated neural circuits, particularly in relation to pain, stress, and metabolic regulation. Thus, we conducted a comprehensive review of past and current research on the role of the hypothalamus in mediating the therapeutic effects of acupuncture.

## 1. Introduction

Acupuncture, a traditional oriental medicine, has been utilized for centuries to treat various disorders [1,2,3,4]. This technique involves the insertion of fine needles at specific points on the body, believed to modulate the flow of vital energy, or “Qi”, thereby restoring balance and promoting healing [5,6]. In recent years, acupuncture has gained significant attention in the scientific community, particularly within the fields of neuroscience for its potential to influence brain function and neurochemical pathways [7,8,9].

The hypothalamus, a small yet critical region in the brain, plays a pivotal role in maintaining homeostasis by regulating a wide array of physiological processes, including stress responses, energy balance, and pain modulation [10,11]. Additionally, the lateral hypothalamic area (LH), a subregion within the hypothalamus, has been implicated in motivated behaviors [12,13]. Emerging evidence suggests that acupuncture may exert its therapeutic effects by modulating the activity of the hypothalamus and its associated neural circuits, particularly in relation to pain, stress, and metabolic regulation [14,15].

Given the complexity of brain functions and the multifaceted nature of acupuncture’s effects, it is essential to explore the underlying mechanisms that link acupuncture to hypothalamic regulation. Understanding these connections can provide valuable insights into how acupuncture contributes to the treatment of various conditions, such as chronic pain and stress-related disorders.

This review aims to synthesize current research on the interactions between acupuncture and the hypothalamus, with a particular focus on the LH. By examining the previous studies that investigated acupuncture’s influence on pain modulation, stress, and anxiety, this review will highlight the potential pathways through which acupuncture may exert its therapeutic effects. Furthermore, this paper will discuss the implications of these findings for future research and clinical applications, offering a brief overview of the role of the hypothalamus in acupuncture’s effects.

## 2. Hypothalamus and Lateral Hypothalamic Area

The hypothalamus, located in the sagittal section of the brain, is noted for its distinct rhombus-shaped appearance, extending from the optic chiasm and lamina terminalis [16]. This area of the brain establishes the foundations of the third ventricle walls and actively secretes hormones responsible for the heart rate, hunger, body temperature, and moods/feelings [17,18]. The primary goal of the hypothalamus is to help the human body retain homeostasis—the activity of cells that maintain internal stability—thus allowing the body to withstand foreign stimuli [19,20]. One notable component of the hypothalamus is the lateral hypothalamic area (LH), which has been identified as a neuroanatomical substrate playing a critical role in motivated behavior in humans and animals [21,22]. The LH has a less anatomic definition than the hypothalamus, as it consists predominantly of neurons and fibers [22,23]. Despite making up only 3% of the total brain tissue, the hypothalamus largely controls broader homeostatic functions, while the LH is responsible for arousal and energy homeostasis [22,24].

In the context of animal behavior, LH plays important roles in survival-related behaviors [25]. When the LH is stimulated electrically, patterns in appetite, drinking, and gnawing are prevalent in sated animals [26,27,28]. It is important to note that, because this assumption was proven based on tests that were conducted on a variety of animal species as opposed to a homogenous sample, the electrical stimulation produced vastly different neural responses [29,30]. The consensus of this study is that the stimulation of LH results in different primitive reactions in animals; however, such an outcome does not entail a difference in the stimulation region within the LH but rather the result of response patterns developed during each stimulation trial [31,32]. Environmental factors, such as temperature and malady, could also have played a role in this study. The stimulation of the region prompted behaviors such as feeding, drinking, gnawing, or attacking another animal [21,33,34]. These particular reactions are likely a response to the tedious nature of the procedure, where the stimulation of the LH is applied to the animals repeatedly, thus making the subject lose control of their primal instincts [34,35,36]. This is especially evident when considering that the electrical LH stimulation provokes food-reinforced responses, parallel to reinforced behaviors that occur when the test subjects are fully deprived of food and water [21,34,37]. Thus, the study suggests that stimulation-induced eating and drinking are responsive to both low and high stimulation frequencies; the stimulation of the LH ultimately elicits considerable hunger and thirst responses [21,34,38].

The LH is also notable in its physical composition, consisting of vesicular glutamate transporter type 2 mRNA expressions and GABAergic neuronal markers [39]. This suggests that LH neuronal subpopulations synthesize glutamate, an excitatory neurotransmitter, as VGlut2 is primarily responsible for filling the synaptic vesicles of excitatory neurons with glutamates in the central nervous system [40,41]. GABAergic neurons are largely segregated from VGlut2 cells in the LH, aiding the transition from potassium efflux of the action potential in the axon hillock to the restoration of equilibrium in the K-channel [41,42]. LH neurons secrete neuropeptides such as orexin (also called as hypocretin), melanin-concentrating hormones, galanin, and neurotensin [43,44,45]. These neuropeptides are likely to play a role in feeding and reward behaviors in animals as well as metabolism [45,46,47]. The orexin neuropeptides are restricted to the lateral hypothalamic area [48]. As one of primary agents for feelings of arousal, Orx neurons play a proper role in reward behaviors [48,49]. The melanin-concentrating hormone-producing neurons are also found predominantly in the LH, consisting of markers for glutamic acid decarboxylase or glutamate (VGlut2) [50,51]. This suggests that inhibitory and excitatory cells are defining features of MCH neurons in the LH. According to a study by Ludwig DS et al., the overexpression of melanin-concentrating hormones directly leads to hyperphagia and obesity in mice, while the lack of MCH leads to a drastic improvement in health conditions and physique in mice [46,52]. It is also important to distinguish that, unlike Orx neurons, MCH neurons promote REM (rapid eye movement) sleep instead of arousal [43,53,54]. Neurotensin-producing neurons are believed to play a significant role in negative energy balance and the lack of neurotensin neurons correlates to hyperphagia and obesity; MCH neurons show the opposite effect in mice [22,45].

Neurotensin neurons have more overlap with galanin-expressing neurons as opposed to Orx and MCH neurons, which suggests that the bulk of LH neurons that were targeted in the Vgat-ires-Cre line are likely to be neurotensin neurons [45,55]. The LH receives inhibitory and exhibitory inputs from cortical and subcortical structures [45,55]. When the medial prefrontal cortex undergoes electrical stimulation, polysynaptic and monosynaptic activity patterns are prevalent in the neurons of the lateral hypothalamic area [56]. Neuromodulators, such as serotonin, dopamine, and norepinephrine, are released in the LH to regulate “circuit dynamics” [57,58]. Moreover, there exist various intra-hypothalamic connections where regions such as the arcuate nucleus, periventricular hypothalamus, and ventral medial hypothalamus contribute inputs to the LH [59,60]. When stimulating the arcuate AgRP (Agouti-Related Peptide)-LH or paraventricular GABA-LH pathways optogenetically, feeding behavior can be induced [61,62].

### Mechanisms of Acupuncture Effects

The mechanisms by which acupuncture exerts its effects on the hypothalamus are summarized in Table 1.

1. **Pain Modulation**

Pain serves as a response to a discomforting stimulus that signals the victim to withdraw or protect themselves from the stimulus, particularly referring to a breakage in tissue [63,64]. Pain relief is a negative reinforcement of pain and activates the reward system of the brain [65]. The practice of acupuncture is known to relieve chronic and prolonged physical or mental pain so that the patient does not succumb to the feelings of discomfort several hours after the end of the procedure [66,67]. In addition to minor physical pain or complications, acupuncture has proven to significantly improve cognitive impairment as well as depression, anger, or fear [68,69]. The midbrain limbic reward network, better known as the mesolimbic dopamine (DA) system, contains the hypothalamus and serves as a large contributor to the reward system [70]. The DA circuit was initially believed to extend from the ventral tegmental system to the nucleus accumbens, thus constituting the basic midbrain reward system [71,72]. However, recent studies have shown that the reward loop is considerably more intricate and complex. For example, the nucleus accumbens (NAc) receives neural DA projections from the ventral tegmental area (VTA) of the midbrain, while the NAc receives projections from the amygdala, prefrontal cortex, and anterior cingulate cortex to stimulate reward mechanisms in the brain [73]. The mesolimbic dopamine system also interacts with the hypothalamus through locomotion and feeding behaviors to program metabolism and reward [13,74,75]. Acupuncture analgesia, in particular, activates brain reward systems such as the brain striatal system, which is primarily responsible for pleasure [76]. Acupuncture produces an analgesic effect by modulating the limbic lobe–limbic lobe–neocortex network system, specifically the limbic cortex, subcortical structures, and the hypothalamus [77,78,79]. One functional magnetic resonance imaging study showed that, by increasing the patient’s expectation of acupuncture, the therapeutic effect of knee osteoarthritis pain increased, thus indicating that the areas of the brain associated with pleasure and pain relief, namely the anterior cingulate gyrus and lateral hypothalamic area, are activated during acupuncture [80,81].

Electroacupuncture (EA) is a form of acupuncture that involves two needles as opposed to the one needle used in standard acupuncture practices and is intended to apply more stimulation to acupoints on the body; an electric current passes through the needles throughout the treatment, hence the distinction from standard acupuncture therapy [82]. Electroacupuncture activates the brain reward system and increases the level of 5-hydroxytryptamine in the NAc of conscious rats, which suggests that it can potentially be used to treat depression and other types of mental pain in human beings [83]. The lateral hypothalamic area stores orexin neurons for the NAc, and the pathways for this process can be activated by EA-induced rewards of pain relief [84]. Orexin microinjection in the NAc shell activates NAc dopaminergic and GABAergic cells, while the injection of orexin-A antagonist into the NAc minimizes the conditioned place preference (CPP) for morphine [84]. These findings are indicative of the significance of the LH in acupuncture pain modulation; orexin release in the NAc allows reward behaviors to take effect.

While the precise neural mechanisms underlying acupuncture’s analgesic effects are not yet fully elucidated, evidence indicates that both standard and electroacupuncture may influence reward and pain pathways involving the mesolimbic dopamine system, the hypothalamus, and the lateral hypothalamic area. Notably, orexin release in the NAc appears to mediate aspects of acupuncture’s pain relief and reward modulation. Although these findings are promising, they warrant further rigorous research—particularly in diverse clinical settings—to confirm broader applicability and long-term safety. As our understanding of acupuncture’s integrative effects deepens, it may evolve into a valuable complementary approach for managing chronic pain and associated psychological distress.

2. **Stress and Anxiety Reduction**

Recent studies have demonstrated that acupuncture can significantly impact drug addiction by modulating the hypothalamus and its associated neural pathways [85,86,87,88]. The LH plays a pivotal role in the brain’s reward system, which is heavily implicated in addictive behaviors [87,89]. The mesolimbic dopamine (DA) system, connecting the VTA to the NAc, is central to the brain’s reward circuitry and strongly linked to drug addiction. Acupuncture has been shown to modulate dopamine release within this system, particularly through its effects on the hypothalamus and LH [87]. For instance, in cocaine-treated rats, acupuncture applied to the HT7 (Heart 7) or *Shenmen* acupoints, located at the ulnar end of the transverse crease of the wrist, resulted in the suppression of cocaine-induced dopamine release in the NAc. This effect appears to be mediated by the LH, which can regulate dopamine signaling in the reward pathway. By reducing dopamine in the NAc, acupuncture may diminish the reinforcing effects of drugs and attenuate drug-seeking behaviors and the risk of relapse [89,90].

Orexin neurons located in the LH are crucial regulators of reward behaviors, including those related to drug addiction [84]. Acupuncture has been shown to modulate orexin pathways, influencing addiction-related behaviors. Acupuncture’s influence on hypothalamic activity extends to the regulation of stress responses, which are closely linked to addiction relapses [91,92]. By modulating neurotransmitter systems like dopamine and orexin in the LH, acupuncture not only reduces the pleasure associated with drug use but also helps alleviate withdrawal symptoms [87,90]. Research indicates that acupuncture influences the hypothalamic–pituitary–adrenal (HPA) axis, affecting the release of corticotropin-releasing hormone (CRH) and neuropeptide Y (NPY), thereby modulating stress responses [91,92]. For instance, a study demonstrated that electroacupuncture at the ST36 (Stomach 36) or called as *Zusanli*, acupoint in rats subjected to cold stress resulted in significant reductions in plasma ACTH and corticosterone levels, as well as decreased hypothalamic CRH levels, compared to stressed controls [93]. Additionally, electroacupuncture prevented stress-induced elevations in adrenal NPY mRNA expression. Through its multifaceted effects on the hypothalamus and related neural pathways, acupuncture offers a promising complementary approach for the treatment of drug addiction. By modulating key neurotransmitter systems and stress responses, acupuncture can potentially reduce drug-seeking behaviors, alleviate withdrawal symptoms, and prevent relapse, thereby contributing to more effective addiction management strategies.

**Table 1:** Acupuncture’s mechanism via the hypothalamus.

Mechanism	Role of Hypothalamus and LH	Effect of Acupuncture	Result or Effect	Main Neurotransmitters and Pathways	References
Pain Modulation	- Hypothalamus is involved in pain modulation and reward system - LH participates in reward behavior via orexin neurons	- Modulates neural circuits associated with the hypothalamus to alleviate pain - Activates the mesolimbic dopamine system	- Pain relief and activation of the reward system - Reduction in discomfort associated with pain	- Dopamine (DA) - Orexin - Mesolimbic dopamine system	[72,73,75]
Stress and Anxiety Reduction	- Hypothalamus plays a key role in stress response and HPA axis regulation - LH is involved in stress responses via orexin neurons	- Regulates the HPA axis to control stress hormone secretion - Influences the release of CRH and NPY in the hypothalamus	- Decreased levels of stress hormones (adrenocorticotropic hormone (ACTH), cortisol) - Alleviation of stress and anxiety symptoms	- Corticotropin-releasing hormone (CRH) - Neuropeptide Y (NPY) - Enkephalin - Serotonin	[9,85,92]
Modulation of Addiction Behavior	- LH plays a central role in the reward system - Orexin neurons regulate reward behaviors related to addiction	- Modulates dopamine release via the hypothalamus and LH - Acupuncture at HT7 reduces dopamine levels in the NAc	- Decreased reinforcing effects of drugs - Reduction in drug-seeking behavior and relapse - Alleviation of withdrawal symptoms	- Dopamine (DA) - Orexin - HPA axis - CRH and NPY	[79,81,82,85]

Stress is properly defined as an unbalanced interaction between a “deforming force against a resisting force” [94]. Negative stress, stimulated by negative feedback, is referred to as distress, whereas positive stress, stimulated by positive feedback, is referred to as eustress [95,96]. Endocrinologist Hans Selye interprets stress as a “whole body” response, as various parts of the body work together to elicit stress reactions; there is no one specific region of the body that is particularly specialized for handling stress [97,98]. As a result, Selye coins the effects of stressors on the body as the “general adaptation syndrome model”, which in theory consists of an alarm reaction, stage of resistance, and stage of exhaustion [98]. Acupuncture is known to significantly reduce the effects of the three stages by reversing the fight or flight response and promoting the relaxation response [94]. Both the fight or flight and reaction responses are stimulated by the autonomic nervous system, with the hypothalamus playing a key role in regulating autonomic functions [18]. Acupuncture can lower stress by providing the central nervous system and plasma with an influx of enkephalin, which are endogenous opioid peptides primarily responsible for regulating mood and emotions, containing antidepressant and anxiety-relieving properties [94,99]. The hypothalamic paraventricular nucleus (PVN) contains the paravicellular subnucleus responsible for the secretion of stress hormones, such as corticotrophin-releasing hormones, thyrotropin-releasing hormones, and enkephalins, which are then activated by acupuncture therapy [17,100]. Acupuncture also increases the plasma levels of beta-endorphin and ACTH by releasing larger concentrations of the said hormones from the anterior lobe of the pituitary gland [101,102]. Serotonin is a chemical that regulates mood and is strongly associated with feelings of ecstasy and sexual arousal [103,104]. During the practice of acupuncture, there is a substantial influx of serotonin in the central nervous system, which explains the ease of stress and anxiety in patients who undergo acupuncture treatment [83,105]. Neurons containing serotonin supply corticotropin-releasing hormones in the paraventricular nucleus of the hypothalamus to coordinate the release of CRH and adrenocorticotropin, which triggers the secretion of glucocorticoids from the adrenal cortex [106,107]. This exchange between the serotonin and hypothalamus plays a key role in eliciting stress relief responses, as seen in acupuncture therapy.

Taken together, the current data suggest that acupuncture may modulate both addiction-related and stress-regulating pathways via the hypothalamus, lateral hypothalamic area, and broader autonomic and limbic circuits. By influencing important neurotransmitters such as dopamine, orexin, and serotonin, acupuncture could help reduce drug-seeking behaviors, alleviate withdrawal symptoms, and mitigate stress responses. Nonetheless, further studies are necessary to elucidate precise mechanisms and validate clinical out-comes. Approached as a complementary strategy rather than a standalone remedy, acupuncture shows promise for a more comprehensive management of addiction- and stress-related disorders.

3. **Modulation of Addiction Behavior**

Recent studies have demonstrated that acupuncture can significantly impact drug addiction by modulating the hypothalamus and its associated neural pathways [85,86,87,88]. The LH plays a pivotal role in the brain’s reward system, which is heavily implicated in addictive behaviors [87,89]. The mesolimbic DA system, connecting the VTA to the NAc, is central to the brain’s reward circuitry and strongly linked to drug addiction. Acupuncture has been shown to modulate dopamine release within this system, particularly through its effects on the hypothalamus and LH [87]. For instance, when acupuncture was applied to the HT7 point in cocaine-treated rats, it reduced dopamine levels in the NAc via the LH. This reduction in dopamine can potentially decrease the reinforcing effects of drugs, lowering drug-seeking behaviors and the likelihood of relapse. Orexin neurons located in the LH are crucial regulators of reward behaviors, including those related to drug addiction [89,90]. Acupuncture has been shown to modulate orexin pathways, influencing addiction-related behaviors. Acupuncture’s influence on hypothalamic activity extends to the regulation of stress responses, which are closely linked to addiction relapses [91,92]. By modulating neurotransmitter systems like dopamine and orexin in the LH, acupuncture not only reduces the pleasure associated with drug use but also helps alleviate withdrawal symptoms [87,90]. Research indicates that acupuncture influences the hypothalamic–pituitary–adrenal (HPA) axis, affecting the release of corticotropin-releasing hormone (CRH) and neuropeptide Y (NPY), thereby modulating stress responses [91,92]. For instance, a study demonstrated that electroacupuncture at ST36 acupoint in rats subjected to cold stress resulted in significant reductions in plasma ACTH and corticosterone levels, as well as decreased hypothalamic CRH levels, compared to the stressed controls [93]. Additionally, electroacupuncture prevented stress-induced elevations in adrenal NPY mRNA expression. Through its multifaceted effects on the hypothalamus and related neural pathways, acupuncture offers a promising complementary approach for the treatment of drug addiction. By modulating key neurotransmitter systems and stress responses, acupuncture can potentially reduce drug-seeking behaviors, alleviate withdrawal symptoms, and prevent relapse, thereby contributing to more effective addiction management strategies.

In conclusion, it can be stated that acupuncture has the potential of helping in the management of addiction through the hypothalamus, lateral hypothalamic area, and the associated reward and stress pathways. However, there is a need for more large-scale clinical trials in order to determine the effectiveness of the findings as well as the sustainability of the results. If confirmed, acupuncture can be incorporated into various treatment protocols as a beneficial component in the treatment of addiction as it may help in decreasing drug use, manage withdrawal symptoms, and promote sustained recovery.

## 3. Conclusions

The primary objective of this review was to explore how acupuncture therapy relates to hypothalamic function, with a particular focus on the lateral hypothalamic area. In this paper, we described acupuncture’s potential impact on pain modulation, stress and anxiety responses, and addictive behaviors. The accumulating evidence supports the notion that acupuncture exerts multifaceted influences on hypothalamic pathways and can generate analgesia, stress relief, and anti-addictive effects. Despite encouraging results—such as reduced pain, improved stress tolerance, and beneficial effects on addiction circuits—several critical gaps remain. Most studies rely on relatively small or heterogeneous samples, limiting the generalizability of their findings. Additionally, standardized protocols for acupuncture (e.g., point selection, frequency of treatment, and needle stimulation parameters) are not uniformly established, complicating direct comparisons across studies. Careful interpretation and further scientific scrutiny are warranted, but the potential benefits of acupuncture highlight its value as both a stand-alone and adjunct therapeutic strategy in modern medicine.

Nonetheless, the accumulating body of evidence supports the notion that acupuncture exerts multifaceted influences on hypothalamic pathways and associated neural circuits. As more rigorous investigations address current limitations—by employing standardized methodologies, larger cohorts, and better controls—acupuncture may emerge as a clinically robust, integrative intervention for a variety of conditions tied to hypothalamic dysregulation. Careful interpretation and further scientific scrutiny are warranted, but the potential benefits of acupuncture highlight its value as both a stand-alone and adjunct therapeutic strategy in modern medicine.

## 4. Future Perspectives

Although current research has started to demonstrate how acupuncture can affect the hypothalamic pathways, to have a clearer scientific view, there should be several strategic approaches. First, there is a need for more large-scale clinical trials with good randomization and blinding to establish the efficacy in the general population. Second, the use of multiple neuroimaging techniques including fMRI, positron emission tomography, and magnetic encephalogram, as well as the molecular investigations like proteomics and transcriptomics, can help to determine the specific brain networks and signaling pathways involved in acupuncture. Third, if the treatment parameters, which include acupoint choice, depth of needle insertion, stimulation frequency, and duration of the session, could have been standardized, this would have enhanced the reproducibility and the comparability of the results across the different studies. Lastly, the conduction of acupuncture with other new therapeutic approaches like biofeedback, neuromodulation agents, or pharmacotherapy may provide potential additive benefits for diseases including chronic pain, metabolic syndrome, and psychiatric disorders. Through the effective implementation of the above recommendations, future research will be able to enhance the scientific rationale supporting acupuncture and provide a platform for the development of more efficient and specific clinical applications.

## Data Availability

No new data were created or analyzed in this study. Data sharing is not applicable to this article.

## Abbreviations

ACTH	Adrenocorticotropic Hormone
AgRP	Agouti-Related Peptide
CPP	Conditioned Place Preference
CRH	Corticotropin-Releasing Hormone
DA	Dopamine
EA	Electroacupuncture
GABA	Gamma-Aminobutyric Acid
HPA	Hypothalamic-–Pituitary-–Adrenal
HT7	Shenmen (Heart 7)
LH	Lateral Hypothalamus
MCH	Melanin-Concentrating Hormone
mRNA	Messenger Ribonucleic Acid
NAc	Nucleus Accumbens
NPY	Neuropeptide Y
VGlut2	Vesicular Glutamate Transporter Type 2
VTA	Ventral Tegmental Area
